# YY1 Promotes Telomerase Activity and Laryngeal Squamous Cell Carcinoma Progression Through Impairment of GAS5-Mediated p53 Stability

**DOI:** 10.3389/fonc.2021.692405

**Published:** 2021-08-23

**Authors:** Xudong Wei, Fenglei Liu, Xuelian Jiang, Xiaoyan Xu, Tianhao Zhou, Chengfang Kang

**Affiliations:** ^1^Department of E.N.T., Gansu Provincial Hospital, Lanzhou, China; ^2^The First School of Clinical Medicine, Lanzhou University, Lanzhou, China; ^3^The First School of Clinical Medicine, Gansu University of Chinese Medicine, Lanzhou, China

**Keywords:** Yin Yang 1, LncRNA growth arrest-specific 5, p300, p53, laryngeal squamous cell carcinoma, telomere length, telomerase activity

## Abstract

Yin Yang 1 (YY1) is a key transcription factor that exerts functional roles in the cell biological process of various cancers. The current study aimed to elucidate the role and mechanism of YY1 in laryngeal squamous cell carcinoma (LSCC). YY1 mRNA and protein expression in human LSCC cell lines was detected by RT-qPCR and Western blot analysis. An interaction of YY1, GAS5, and p53 protein stability was predicted and confirmed by bioinformatics, ChIP, Co-IP, RIP, and FISH assays. Following loss- and gain-function assays, LSCC cell proliferation, colony formation, cell cycle, telomere length and telomerase activity were evaluated by CCK-8 assay, colony formation assay, flow cytometry, and PCR-ELISA, respectively. Nude mice were xenografted with the tumor *in vivo*. LSCC cell lines presented with upregulated expression of YY1, downregulated GAS5 expression, and decreased p53 stability. YY1 inhibited the expression of GAS5, which in turn recruited p300 and bound to p53, thus stabilizing it. Moreover, YY1 could directly interact with p300 and suppressp53 stability, leading to enhancement of cell proliferation, telomere length and telomerase activity *in vitro* along with tumor growth *in vivo*. Collectively, YY1 can stimulate proliferation and telomerase activity of LSCC cells through suppression of GAS5-dependent p53 stabilization or by decreasing p53 stability *via* a direct interaction with p300, suggesting that YY1 presents a therapeutic target as a potential oncogene in LSCC development and progression.

## Introduction

Laryngeal squamous cell carcinoma (LSCC) is a common malignant tumor occurring in the respiratory tract, which severely affects quality of life of the patients by compromising the ability to talk, breathe, and swallow (1). People living in areas with serious air pollution and those who smoke, drink excessively, and have a pro-inflammatory diet are at elevated risk for LSCC (2, 3). Current treatment approaches for this disease consist of surgery, radiation, chemotherapy, and biotherapy, but the overall outcomes remain unsatisfactory, possibly due to alterations in treatment patterns and treatment-associated toxicities (4). Recently, the activation of telomerase has been highlighted to be a prerequisite for the tumorigenesis of LSCC that is associated with cell proliferative potential (5). Therefore, we are motivated to identify specific molecular signatures that inhibit telomerase activation, which might serve as suitable therapeutic targets for LSCC.

Yin Yang 1 (YY1) is a transcription factor that is often overexpressed in cancers, and which is involved in the regulation of tumor cell growth, proliferation, migration, and metastasis (6). YY1 is a useful molecular target as a potential oncogene in head and neck squamous cell carcinomas (7) and is especially implicated in laryngeal cancer development and progression, owing to its promoting effects on the malignant characteristics of cancer cells (8). YY1 can directly bind to the promoter region of multiple long non-coding RNAs (lncRNAs) to regulate their expression (9, 10). LncRNAs, a group of non-protein-coding transcripts exceeding 200 nt in length, participate in various vital biological processes by regulating gene expression (11), and are thus candidates for diagnostic and prognostic LSCC biomarkers (12). The lncRNA growth arrest-specific 5 (GAS5) is related to the clinicopathological features of LSCC patients, and its upregulation hinders LSCC progression *via* negative regulation of microRNA (miR)-21 (13), indicating its potential as a biomarker and potential target for LSCC therapy. In addition, the regulatory role of GAS5 on p53 expression has been attributed to derivation of small nucleolar RNA (snoRNA) (14). p53 is an extensively studied tumor suppressor gene that regulates cell cycle progression, apoptosis, senescence, and metabolic adaptation, and which general prevents carcinogenesis (15). Activated p53 following diaphanous related formin 1 (DIAPH1) knockdown can stimulate the apoptosis of LSCC cells (16). Additionally, increased p53 expression is capable of reducing telomere length and preventing telomerase activity, thus retarding cancer progression (17). In the light of the aforementioned data, we thus hypothesize that YY1 may affect LSCC progression *via* regulation of the GAS5-mediated p53 expression. To test this prediction, we first performed bioinformatics analysis, and then employed an array of functional assays to expound upon the mechanism of YY1 in regulating telomerase activity and resultant effects on LSCC progression.

## Materials and Methods

### Ethics Statement

The current study was approved by the Ethics Committee of Gansu Provincial Hospital and conducted in strict accordance with the Guide for the Care and Use of Laboratory Animals published by the US *National Institutes of Health*. All efforts were made to minimize the number and suffering of the included animals.

### Cell Culture

Human LSCC cell lines (SNU899 and SNU1076) were obtained from Shanghai Chuanqiu Biotechnology Co., Ltd. (Shanghai, China), while NP-69 and 293T cells were purchased from the Chinese Academy of Sciences (Shanghai, China). All cell lines were subjected to mycoplasma testing every three months and confirmed to be free of infection before use. The cells were cultured in Roswell Park Memorial Institute (RPMI)-1640 medium containing 10% fetal bovine serum (Invitrogen Inc., Carlsbad, CA, USA), 100 units/ml penicillin and 100 μg/ml streptomycin in a wet incubator with 5% CO_2_ at 37°C.

### Cell Transfection

GAS5 antisense oligonucleotide (ASO) was designed and synthesized by Ribobio (Guangzhou, China). Short hairpin RNAs (shRNAs) targeting p53 and YY1 were designed and synthesized by Biotend Biotechnology (Shanghai, China) and cloned into the adenovirus pFH-l plasmid vector (Shanghai Genechem Co., Ltd., Shanghai, China). Cells were then transfected with the aforementioned plasmids using Lipofectamine 3000 and RNAiMAX reagents (Invitrogen). At 24 h after transfection, the corresponding experiment was carried out. The overexpression of YY1 fragment used in xenograft tumor in nude mice was produced by PCR amplification with human YY1 as the template, and then cloned into the pAdTrack adenovirus vector.

### Cell Counting Kit-8 Assay

After transfection, cells in the exponential growth phase were trypsinized and prepared into a cell suspension. The suspension was then seeded into a 96-well plate at a density of 1 × 10^5^ cells/well, and each well was added with 200 μl of medium and labeled, followed by incubation with 5% CO_2_ at 37°C for 24, 48, and 72 h. CCK-8 solution was incubated in the 96-well plate, with 10 μl per well at 37°C for 4 h. Finally, the optical density value was measured at 450 nm using a microplate reader (Thermo Fisher Scientific, San Jose, CA, USA).

### Colony Formation Experiment

The cells were seeded into a six-well plate at a density of 300 cells/well, with three replicate wells set in each group. After 14 days of culture, the cells were washed three times with phosphate buffered saline (PBS). Then, the cells were fixed with 4% paraformaldehyde in PBS and stained with 0.1% crystal violet for 30 min. Following the excess dye removal, the colony formation of cells was observed under an inverted microscope to calculate the colony formation rate.

### Flow Cytometry

Cells were collected and then fixed overnight with precooled 70% (v/v) ethanol at 4°C. The cells were then resuspended in PBS containing RNaseA (20 μg/ml) and propidium iodide (20 μg/ml) for 20 min, and the DNA was stained for 20 min. The histogram of DNA content was obtained by gating the cell population on the fluorescence intensity/scatter plot, whereupon the Watson Pragmatic model in the FlowJo software (version 10.2) was applied to fit the obtained histogram to quantify the cell proportion per cell cycle.

### Xenograft Tumor in Nude Mice

Forty-eight BALB/c nude mice (4–5 weeks old, weighing 15–30 g; from the Experimental Animal Center of the Chinese Academy of Sciences, Shanghai, China) were raised for 1 week under specific pathogen free (SPF) conditions at 18–23°C, humidity of 50–60%, and a 12-h light/dark cycle with free access to food and drinking water. Subsequently, the mice were injected with SNU899 or SNU1076 cells transfected with sh-negative control (NC) + sh-NC (n = 8), sh-YY1 + sh-NC (n = 8) and sh-YY1 + sh-p53 (n = 8). The cells (1 × 10^7^ cells/ml) were subcutaneously injected into the nude mice. The tumor volume was measured every 7 days and calculated using the formula: tumor volume = length × width^2^ × 0.5. At 42 days after injection, the mice were euthanized by overdose of isoflurane, and the tumor was removed, weighted, and measured. Then tumor growth curves were plotted by treatment group.

### Immunohistochemical Staining

The paraffin-embedded tissues were used for this experiment. The sections were dewaxed and rehydrated. Following 2 min of washing, the sections were immersed in 3% formaldehyde-H_2_O_2_ for 20 min and subjected to antigen retrieval in a warm water bath for 10 min. The sections were blocked with normal goat serum blocking solution at room temperature for 20 min, and then probed with primary antibodies to CD276 (ab109237, 1:2000) and p53 (ab26, 1:1000) overnight at 4°C. The next day, the sections were re-probed with secondary goat anti-rabbit Immunoglobulin G (IgG) at 37°C for 20 min and then added with horseradish peroxidase-labeled streptomyces ovalbumin working solution (0343-10000U, Imunbio (Beijing) Biotechnology Co., Ltd., Beijing, China) for 20 min of reaction at 37°C. Next, the sections were developed with 3, 3′-diaminobenzidine (ST033, Guangzhou Whiga Technology Co., Ltd., Guangzhou, China), counterstained with hematoxylin (PT001, Shanghai Bogoo Biological Technology Co., Ltd., Shanghai, China) for 1 min, and returned to blue in 1% ammonia water. The sections were finally analyzed under a microscope (BX63, Olympus, Japan) in five randomly selected high-power fields from each section, with 100 cells counted in each field. Positive cells < 10% were negative, positive cells < 50% were positive, and positive cells > 50% were strong positive (18).

### Immunofluorescence and Fluorescence *In Situ* Hybridization

FISH assay was performed according to the instructions of the View RNA FISH cell detection kit (Affymetrix, Santa Clara, CA, USA). In short, cells were washed twice with PBS, fixed with 4% formaldehyde for 10 min, and permeabilized with 70% ethanol. Before hybridization, the cells were incubated with the pre-hybridization solution at 37°C for 30 min, and then hybridized with the GAS5 probe (GATCCTACTCGAAAAGAAC) (final concentration of 0.5 ng/μl) in the hybridization solution at 37°C overnight. After hybridization, the cells were washed twice in washing buffer at 37°C, 10 min each. The nuclei were stained with 4′,6-diamidino-2-phenylindole (DAPI) for 5 min and then observed under a LSM780 laser scanning confocal microscope (Carl Zeiss, Inc., Oberkochen, Germany).

### Quantitative (Q)-FISH

In short, telomeres were labeled with the probe (Fasmac, Atsugi, Japan; 5′-CCCTAACCCTAACCCTAA-3′) coupled with Cy3 red fluorescence. The Image-Pro Plus software (version 5.0, Media Cybernetics, MD, USA) was used to capture the microscopic images, and the published NIH image program was applied for analysis. The average fluorescence intensity of telomeres in each nucleus in FISH images was calculated.

### PCR-Enzyme-Linked Immunosorbent Assay

Activity of telomerase was measured using PCR-ELISA (Roche, Germany). In short, cells were incubated with 100 μl cold lysis buffer for 30 min to extract cell telomerase protein and then centrifuged at 4°C and 13,000 rpm for 20 min. The supernatant was then harvested, and the protein concentration was determined by the modified Coomassie protein assay (Bradford method) (Pierce). An aliquot of the supernatant equivalent to 2 µg of protein was mixed in 25 µl of reaction mixture supplemented with P1-TS biotinylated telomerase substrate, P2 primer, nucleotides and Taq DNA polymerase, to a 50 µl total volume. An identical 2 µg protein aliquot for each sample that was heated at 94°C for 10 min served as NC, which was otherwise processed in parallel with the test samples. Telomerase activity was measured to exclude false positive results. A 1 µl aliquot of the kit control solution from telomerase-positive cells served as positive control. Following primer elongation for 23 min at 25°C, the telomeric repeat amplification protocol (TRAP) assay was performed in a thermal cycler (94°C for 30 s, 50°C for 30 s, and 72°C for 90 s, to a total of 30 cycles). Afterwards, a 10 µl aliquot of PCR products was denatured using sodium hydroxide and hybridized to digoxigenin-labeled detection probes specific for the telomeric repeats. The obtained products were then fixed to a streptavidin-coated microtiter plate using the P1-TS biotinylated primers, recognized with antibody against digoxigenin conjugated to peroxidase, and developed using tetramethylbenzidine solution. Besides, a 100 µl aliquot of 5% sulfuric acid solution was added to the sample to halt the reaction, whereupon the absorbance was measured at 450 nm using a microtiter plate reader and expressed as the absorbance of the test sample minus the absorbance of the NC.

### Dual-Luciferase Reporter Assay

A p-300 luciferase reporter gene plasmid was constructed. At 24 h before transfection, cells were plated in a 24-well culture plate at a density of 5 × 10^4^ cells/well and transfected with overexpression (oe)-NC and oe-GAS5, where oe-NC served as internal reference. After transfection for 48 h, the cells were incubated with lysis buffer (160 μl per well, Promega Corporation, Madison, WI, USA) for 15 min, and 25 μl of each cell lysate was taken for luciferase activity determination using a LB96v luminometer (Berthold, Nashua, NH). The average value of the Renilla luciferase activity was obtained from three independent experiments.

### RNA Isolation and Quantification

Total RNA content was extracted from cells using TRIzol reagents (Invitrogen Inc., Carlsbad, CA, USA) and reverse transcribed into cDNA using a one-step reverse transcription quantitative polymerase chain reaction (RT-qPCR) kit (Applied Biosystems, USA). RT-qPCR was performed using a SYBR Premix Ex Taq II PCR kit (Takara Biotechnology Ltd., Dalian, Liaoning, China) on an ABI 7500 instrument (Applied Biosystems, Foster City, CA, USA). The primers used are listed in **Table S1**, which were synthesized by Sangon Biotech Co., Ltd. (Shanghai, China). Due to its stability in all experimental groups, glyceraldehyde-3-phosphate dehydrogenase (GAPDH) was used as the internal control, and fold changes were calculated using the relative quantification (2^-ΔΔCt^ method).

### Western Blot Analysis

Total protein was extracted from cells using M-PER buffer solution (Pierce Biotechnology Inc., Rockford, IL, USA) containing protease inhibitor (Roche, Basel, Switzerland) and Nonidet-P40. The protein concentration was then determined by the modified Coomassie protein assay (Bradford method). Then 50–80 μg of protein was separated using 10% polyacrylamide gel electrophoresis and transferred onto a polyvinylidene fluoride membrane. Next, the membrane was treated with 5–10% skim milk powder or 5% bovine serum albumin (BSA) followed by overnight incubation with primary antibodies against Bcl-2-associated X protein (Bax) (1:1,000, sc-7480), B-cell lymphoma 2 (Bcl-2) (1:1,000, sc-7382), YY1 (1:1,000, sc-7341), telomerase reverse transcriptase (TERT) (1:1,000, sc-393013), p53 (1:1,000, sc-126), p300 (1:2,000, sc-48343), GAPDH (1:1,000, sc-47724), caspase-3 (1:500, ab13847), and cleaved caspase-3 (1:500, ab13847) [all from Abcam Inc. (Cambridge, UK) except GAPDH which was from Santa Cruz Biotechnology, Inc (Santa Cruz, CA, USA)]. The following day, the membrane was re-probed with horseradish peroxidase-labeled secondary antibody for 1 h after Tris-buffered saline Tween-20 (TBST) washing. Afterwards, the membrane was visualized using enhanced chemiluminescence (ECL) reagents (Pierce), and the protein bands were quantified by Image Lab 3.0 software (Bio-Rad, Inc., Hercules, CA, USA).

### Chromatin Immunoprecipitation Assay

Cells seeded in a 10 cm diameter plate were collected and incubated with 37% formaldehyde (243 µl, to a final concentration of 1%, total 9 ml in medium) at 37°C for 10 min to produce DNA–protein cross-linking. Next, 2.5 M glycine (450 µl) to a final concentration of 0.125 M was added to the cells, mixed and allowed to stand at room temperature for 5 min to terminate cross-linking. Then, the cells were lysed with SDS lysis buffer and subjected to ultrasonic treatment with a VCX 750 750-Watt Programmable Ultrasonic Processor, at power of 25%, pulse of 4.5 s, and intervals of 9 s, for a total of 14 cycles, to fragment the cross-linked sample. Cells were centrifuged at 10,000 g for 10 min at 4°C to separate and discard the insoluble matter. Afterwards, 100 µl of the sonicated products was added and mixed with 900 µl ChIP Dilution Buffer and 20 µl of 50× PIC and then with 60 µl Protein A Agarose/Salmon Sperm DNA, followed by spinning at 4°C for 1 h. The samples were centrifuged at 700 rpm for 1 min and the supernatant was collected, 20 µl of which was removed as input, and 20 µl of which was incubated with 1 µl of antibody overnight at 4°C. Thereafter, immune complexes were precipitated and washed, after which DNA samples were recovered and subjected to RT-qPCR analysis. Primers for GAS5 and YY1 were designed for PCR amplification.

### Co-Immunoprecipitation Assay

Cells were rinsed twice with pre-cooled PBS, lysed with pre-cooled RIPA Buffer (1 ml/10^7^ cells, 10 cm diameter culture dish or 150 cm^2^ culture flask, 0.5 ml/5 × 10^6^ cells, 6 cm diameter culture dish, 75 cm^2^ culture flask) and scraped from the culture dish or flask using a pre-cooled cell scraper. The collected cell suspension was transferred to a 1.5 ml Eppendorf (EP) tube and centrifuged at 14,000 g and 4°C for 15 min, whereupon the supernatant was immediately transferred to a new centrifuge tube. Protein A Agarose beads were prepared, and washed twice with PBS to a concentration of 50%. Each 1 ml of total protein sample was added and mixed with 100 μl Protein A Agarose beads (50%) and centrifuged at 14,000 g and 4°C for 15 min. The supernatant was collected into a new centrifuge tube, and the beads were removed, whereupon the protein concentration was measured using the Bradford method. Before this determination, the total protein was diluted at least 10-fold to about 1 μg/μl with PBS to eliminate the interfering effect of detergent carried over from the cell lysate. Next, 100 μl portions of beads were added to immunoprecipitate the antigen–antibody complex by gentle shaking overnight at 4°C. The next day, centrifugation was performed at 14,000 rpm for 5 s, and the Agarose beads-antigen-antibody complex was collected. Following washing, the beads-antigen-antibody complex was suspended in 60 μl 2× loading buffer, mixed gently and heated for 5 min, and the complex centrifuged for harvesting of the supernatant harvested to be used for electrophoresis. The remaining beads were collected and denatured by re-heating for 5 min before electrophoresis.

### RNA Binding Protein Immunoprecipitation Assay

Cells were grouped into the Ad-Ctrl, Ad-GAS5, Ad-GAS5 + C646 (a competitive and selective inhibitor of histone acetyltransferase P300), Ad-Ctrl, Ad-GAS5, and Ad-GAS5 + C646 groups. In brief, cell lysate was centrifuged at 20,000 ×g and 4°C for 10 min, and 925 μl of supernatant was added to the antibody-coated magnetic beads to a final volume of 1 ml. Next, 9.7 μl of the sample was taken as Input and stored at −80°C for later use. The sample was mixed overnight in a vertical mixer at 4°C, centrifuged briefly, and transferred onto the magnetic stand in an ice bath. After standing for 1 min, the supernatant was discarded, and the sample was mixed with 1 ml of pre-cooled IP Buffer, centrifuged and again placed on the magnetic stand in an ice bath. After standing for 1 min, the supernatant was discarded. There were five replicates per sample. The precipitate in the RIP experiment was further digested by proteinase K, and the RNA was extracted for subsequent analysis and identification by reverse transcription.

### Statistical Analysis

Statistical analyses were performed using the SPSS 21.0 software (IBM Corp., Armonk, NY, USA). Measurement data were expressed as mean ± standard deviation. Comparison of data between two groups was conducted by unpaired *t*-test while data comparison among multiple groups was conducted using one-way analysis of variance (ANOVA) followed by Tukey’s *post-hoc* tests with corrections for multiple comparison. Bonferroni-corrected repeated measures ANOVA was used for comparison of tumor volume at varied time points and Bonferroni-corrected two-way ANOVA was used for data comparison of cell proliferation at varied time points. A *p*-value <0.05 was considered to be statistically significant.

## Results

### YY1 Potentiates LSCC Cell Proliferation and Tumor Growth but Inhibits Cell Apoptosis

**Figure 1:**
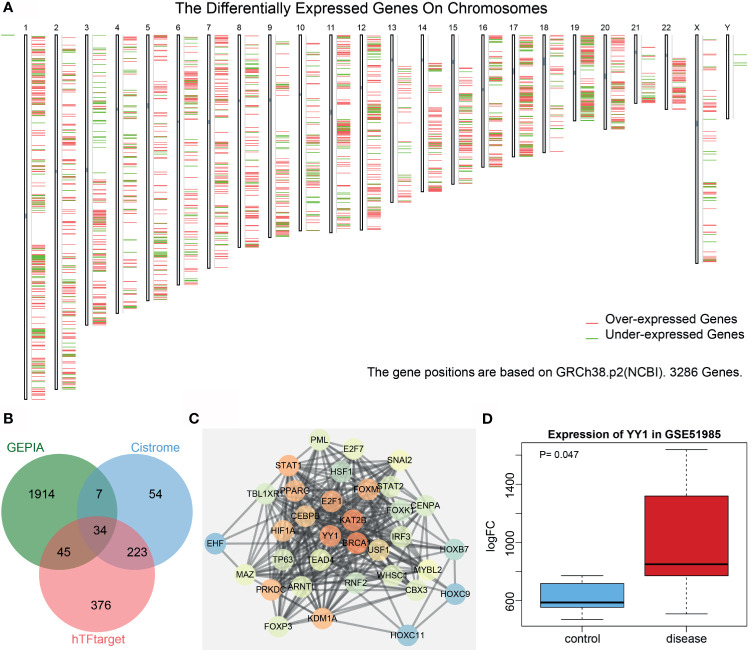
Bioinformatics analysis of the significance of YY1 in LSCC. **(A)** A heatmap of the expression of the first 2,000 differentially expressed genes in each chromosome in LSCC samples from the TCGA database using the GEPIA tool. Red indicates highly expressed genes, and green indicates poorly expressed genes; from the left to the right, there are chromosomes 1–22 and sex chromosome. **(B)** Venn diagram analysis of LSCC-related differentially expressed genes obtained from GEPIA and human transcription factors obtained from hTFtarget and Cistrome. **(C)** Protein interaction network of 34 intersected transcription factors constructed by String. The higher core degree of the gene reflects redder circle, and otherwise, the bluer circle reflects lower core degree. **(D)** A box plot of YY1 expression in the GSE51985 dataset retrieved from the GEO database. Red box represents YY1 expression in LSCC samples and blue box represents YY1 expression in normal samples.

Analysis of the differentially expressed genes in LSCC samples from the Cancer Genome Atlas (TCGA) database (https://www.ncbi.nlm.nih.gov/gds) using the Gene Expression Profiling Interactive Analysis (GEPIA) tool (http://gepia2.cancer-pku.cn/#index) yielded 3,093 genes, of which 2,000 genes showed a significant difference (**Figure 1A**). hTFtarget (http://bioinfo.life.hust.edu.cn/hTFtarget#!/) and Cistrome (http://cistrome.org/) revealed 678 and 318 human transcription factors, respectively, and following intersection analysis, 34 transcription factors with distinctly differential expression were acquired (**Figure 1B**). Then, a protein interaction network of these 34 genes was constructed by String (https://string-db.org/), which showed YY1 to be the third core gene, lying very close to the first two genes (**Figure 1C** and **Table S2**). However, only YY1 has been reported to be associated with LSCC (8). Based on the analysis on the LSCC-related GSE51985 dataset retrieved from the Gene Expression Omnibus (GEO) database (https://portal.gdc.cancer.gov/), YY1 proved to be significantly upregulated in LSCC (**Figure 1D**). We thus selected YY1 for further analysis.

**Figure 2:**
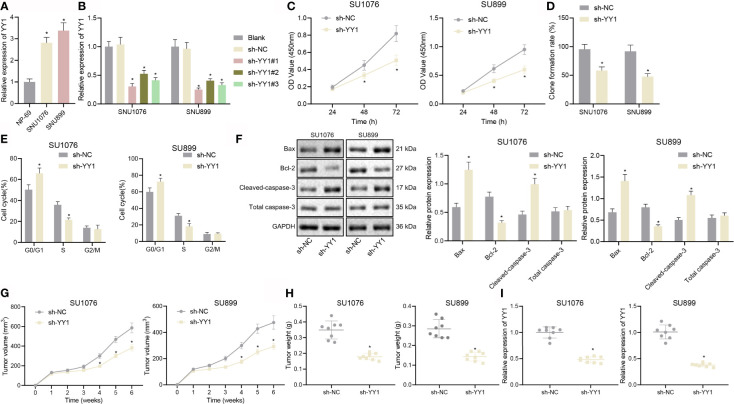
YY1 promotes LSCC cell proliferation and tumor growth but inhibits LSCC cell apoptosis. **(A)** mRNA expression of YY1 in NP-69, SNU1076, and SNU899 cells determined by RT-qPCR, * indicates *p* < 0.05 compared with NP-69. **(B)** mRNA expression of YY1 in SNU1076 and SNU899 cells transfected with sh-YY1#1, sh-YY1#2, and sh-YY13 determined by RT-qPCR. **(C)** Proliferation of SNU1076 and SNU899 cells measured by CCK-8 assay. **(D)** Colony formation of SNU1076 and SNU899 cells measured by colony formation assay. **(E)** Cell cycle distribution measured by flow cytometry in SNU1076 and SNU899 cells. **(F)** Western blot analysis of Bax, Bcl-2, and cleaved caspase-3 proteins in SNU1076 and SNU899 cells, normalized to GAPDH. **(G)** Tumor volume curve of mice. **(H)** Tumor weight of mice. **(I)** mRNA expression of YY1 in tumor tissues of mice determined by RT-qPCR, * indicates *p* < 0.05 compared with sh-NC. Data are shown as mean ± standard deviation of three technical replicates. Data between two groups were compared by unpaired *t*-test while those among multiple groups at varied time points were compared by two-way ANOVA or repeated measures ANOVA with Bonferroni *post-hoc* tests. n = 8 mice in each treatment group.

Next, we attempted to elucidate the role of YY1 in LSCC development. RT-qPCR results showed much higher expression of YY1 in human LSCC cell lines SNU1076 and SNU899 than in normal nasopharyngeal epithelial cells (NP-69) cells (**Figure 2A**). SNU1076 and SNU899 cells transfected with sh-YY1#1, sh-YY1#2 and sh-YY1#3 exhibited a decline of the YY1 mRNA expression, among which sh-YY1#1 had the best silencing effect (**Figure 2B**) and was thus used for the subsequent experiments. Moreover, the results of CCK-8 and colony formation assays revealed a reduction of cell proliferation and colony formation following YY1 silencing (**Figures 2C, D** **)**. Flow cytometric analysis showed more cells arrested in the G0/G1 phase and fewer cells arrested in the S phase, while there was no difference in the G2/M phase-arrested cells (**Figure 2E**). Meanwhile, YY1 silencing resulted in an increased expression of the pro-apoptotic proteins Bax and cleaved caspase-3, but a downward trend in anti-apoptotic protein Bcl-2 expression (**Figure 2F**). Subsequently, we constructed a nude mouse xenograft tumor model, and showed that tumor volume and weight were relatively decreased in mice treated with sh-YY1 (**Figures 2G, H** **)**. We further confirmed that expression of YY1 was diminished in tumor tissues of the mice treated with sh-YY1 (**Figure 2I**). In conclusion, YY1 could promote LSCC cell proliferation but inhibit apoptosis *in vitro*, thus promoting tumor growth *in vivo*.

### YY1 Advances Telomerase Activity and Proliferation but Inhibits Apoptosis of LSCC Cells by Negatively Regulating GAS5

**Figure 3:**
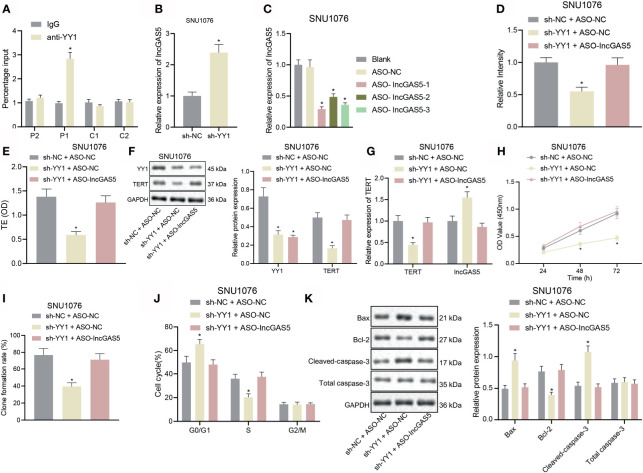
YY1 boosts telomerase activity and proliferation but inhibits apoptosis of LSCC SNU1076 cells by suppressing GAS5. **(A)** ChIP-qPCR was used to detect the binding of YY1 to GAS5 promoter. Primers were designed for P1 and P2 upstream of YY1 and C1 and C2 downstream of YY1. qPCR detected ChIP enriched DNA, * indicates *p* < 0.05 compared with sh-NC + ASO-NC. **(B)** GAS5 expression determined by RT-qPCR in SNU1076 cells transfected with sh-YY1,* indicates *p* < 0.05 compared with sh-NC. **(C)** Knockdown efficiency of GAS5 confirmed by RT-qPCR in SNU1076 cells, * indicates *p* < 0.05 compared with ASO-NC. **(D)** Telomerase length in cells detected by PCR-ELISA. **(E)** Telomerase activity in cells. **(F)** Western blot analysis of YY1 and TERT proteins in SNU1076 cells, normalized to GAPDH. **(G)** mRNA expression of TERT and GAS5 in SNU1076 cells determined by RT-qPCR. **(H)** SNU1076 cell proliferation measured by CCK-8 assay. **(I)** Colony formation of SNU1076 cells measured by colony formation assay. **(J)** SNU1076 cell cycle distribution measured by flow cytometry. **(K)** Western blot analysis of Bax, Bcl-2 and cleaved caspase-3 proteins in SNU1076 cells, normalized to GAPDH, * indicates *p* < 0.05 compared with sh-NC + ASO-NC. Data are expressed as mean ± standard error of the mean. Data are shown as mean ± standard deviation of three technical replicates. Data between two groups were compared by unpaired *t*-test while those among multiple groups were compared by one-way ANOVA with Tukey’s *post-hoc* tests. Bonferroni-corrected two-way ANOVA was applied for data comparison at various time points.

Through co-expression analysis using MEM (https://biit.cs.ut.ee/mem/index.cgi), YY1 and GAS5 were found to have a significant co-expression relationship (**Supplementary Figure S1A**), and meanwhile, GEPIA analysis showed a significant negative correlation between YY1 and GAS5 in LSCC (**Supplementary Figure S1B**). We thus speculated that YY1 initiated the LSCC by negatively regulating GAS5. For validation, we performed an array of experiments. The results of immunofluorescence and FISH assays showed that YY1 and GAS5 were co-localized in the nucleus of SNU1076 and SNU899 cells (**Supplementary Figure S2A**, **Supplementary Figure S5A**). ChIP-qPCR results showed that the transcription factor YY1 P1 site in SNU1076 and SNU899 cells combined with GAS5 promoter (**Figure 3A** and **Supplementary Figure S2B**). Moreover, RT-qPCR data displayed an upward trend for GAS5 expression in SNU1076 and SNU899 cells with YY1 silencing (**Figure 3B** and **Supplementary Figure S2C**). RT-qPCR also verified the transfection efficiency of ASO-GAS5 in SNU1076 and SNU899 cells due to the reduced GAS5 expression following transfection with ASO-GAS5-1, ASO-GAS5-2, and ASO-GAS5-3. Among these treatments, ASO-GAS5-1 showed the best silencing effect (**Figure 3C** and **Supplementary Figure S2D**) and was therefore selected for the follow-up experiments.

Published documents have confirmed that the promoter mutation upstream of TERT may promote the abnormal activation of telomerase in tumor cells; Cheng et al. found the presence of promoter mutation upstream of TERT in 60–80% of conventional urothelial carcinomas and variant urothelial carcinomas (19). In addition, Weyerer et al. confirmed that TERT promoter mutation may have a significant correlation in the early occurrence of bladder cancer (20). Therefore, it is of great importance to explore whether telomerase is abnormally activated in LSCC. As shown in **Figures 3D, E** and **Supplementary Figures S2E, F**, **Supplementary Figure S5B**, telomere length and telomerase activity were decreased upon YY1 silencing alone, but there were no changes upon concomitant silencing of YY1 and GAS5. RT-qPCR and Western blot analysis further confirmed the aforementioned results that TERT mRNA and protein expression was downregulated in response to YY1 silencing alone, but was elevated following concomitant silencing of YY1 and GAS5 (*p* < 0.05) (**Figures 3F, G** and **Supplementary Figures S2G, H**). In addition, we carried out TERT knockout on SNU1076 cells and SNU899 cells respectively. The knockout efficiency of siTERT was verified by RT-qPCR, and then the expression of TERT protein was detected by Western blot, which proved that the TERT antibody was correct (**Supplementary Figures S3A, B**). There was a downward trend in cell proliferation and colony formation following YY1 silencing alone, whereas opposite results were seen upon simultaneous silencing of YY1 and GAS5 (**Figures 3H, I** and **Supplementary Figures S2I, J**). Additionally, silencing of YY1 led to more cells arrested in the G0/G1 phase and fewer cells arrested in the S phase, while there was no difference in the cells arrested in the G2/M phase. However, silencing of both YY1 and GAS5 restored the trends (**Figure 3J** and **Supplementary Figure S2K**). Furthermore, the results of Western blot analysis of key apoptosis-related proteins illustrated an increase in the expression of pro-apoptotic proteins Bax and cleaved caspase-3 but reduction in the anti-apoptotic protein Bcl-2 expression upon YY1 silencing. These factors were rescued upon dual silencing of YY1 and GAS5 (*p* < 0.05) (**Figure 3K** and **Supplementary Figure S2L**). The aforementioned results confirmed that YY1 could stimulate telomerase activity and proliferation but inhibit apoptosis of LSCC cells *via* downregulation of GAS5.

### GAS5 Promotes p53 Stability to Repress Telomerase Activity and Proliferation but Promote Apoptosis of LSCC Cells

**Figure 4:**
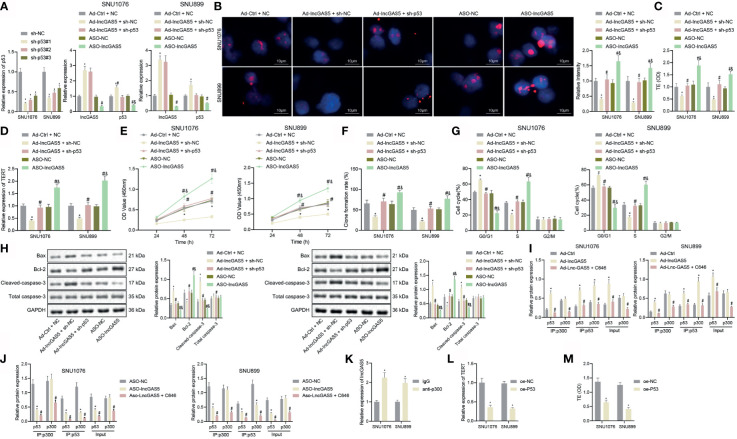
GAS5 impairs telomerase activity and proliferation but promotes apoptosis of LSCC cells by stabilizing p53. **(A)** Knockdown efficiency of p53 as well as p53 stability following GAS5 overexpression or silencing confirmed by RT-qPCR in SNU1076 and SNU899 cells, * indicates *p* < 0.05 compared with sh-NC/Ad-Ctrl + NC, # indicates *p* < 0.05 compared with Ad-GAS5 + sh-NC, and indicates *p* < 0.05 compared with ASO-NC. SNU1076 and SNU899 cells were transfected with GAS5, ASO-GAS5 or GAS5 + sh-p53. **(B)** Telomere length of cells measured by Q-FISH (the left). Blue indicates nucleus, and red indicates telomere, scale bar = 2 μm. mRNA expression of GAS5 and p53 stability in cells determined by RT-qPCR (the right). **(C)** Telomerase activity in cells detected by PCR-ELISA. **(D)** mRNA expression of TERT in cells determined by RT-qPCR. **(E)** Cell proliferation measured by CCK-8 assay. **(F)** Colony formation of cells measured by colony formation assay. **(G)** Cell cycle distribution measured by flow cytometry. **(H)** Western blot analysis of Bax, Bcl-2 and cleaved caspase-3 proteins in cells, normalized to GAPDH, * indicates *p* < 0.05 compared with Ad-Ctrl + NC, # indicates *p* < 0.05 compared with Ad-GAS5 + sh-NC, & indicates *p* < 0.05 compared with ASO-NC. **(I, J)** Protein expression of p300 and p53 stability in the immune complexes detected by Co-IP assay, * indicates *p* < 0.05 compared with Ad-Ctrl/ASO-NC, # indicates *p* < 0.05 compared with Ad-GAS5/ASO-GAS5. **(K)** Interaction between p300 and GAS5 detected by RIP assay, * indicates *p* < 0.05 compared with IgG. **(L)** TERT mRNA expression in cells following p53 overexpression detected by RT-qPCR. **(M)** Telomerase activity in cells following p53 overexpression detected by PCR-ELISA, * indicates *p* < 0.05 compared with oe-NC. Data are shown as mean ± standard deviation of three technical replicates. Data between two groups were compared by unpaired *t*-test while those among multiple groups were compared by one-way ANOVA with Tukey’s *post-hoc* tests. Bonferroni-corrected two-way ANOVA was applied for data comparison at varied time points.

As noted above, TERT promoter mutation and TP53 mutation appear more frequently in advanced tumors, and are often associated with other mutations. Their appearance, especially when coincident, is a sign of aggressiveness (21). In particular, LSCC often has p53 pathway defects. Our present RT-qPCR data showed that p53 stability was reduced in SNU1076 and SNU899 cells following sh-p53#1, sh-p53#2 and sh-p53#3 treatments, with sh-p53#1 exhibiting the best silencing efficiency, leading us to select it for subsequent experiments. Meanwhile, the stability of p53 was elevated following overexpression of GAS5, while the contrary result was noted following silencing of GAS5, and silencing of p53 together with overexpression of GAS5 restored GAS5 expression (**Figure 4A**). Q-FISH results suggested that the telomere length was reduced in cells overexpressing GAS5, while GAS5 silencing increased the telomere length. Both GAS5 overexpression and p53 silencing reduced the telomere length (*p* < 0.05) (**Figure 4B**). In addition, the results of PCR-ELISA revealed a decrease of telomerase activity in the presence of GAS5 overexpression, while GAS5 silencing increased the telomerase activity. However, telomerase activity was reduced in the presence of both GAS5 overexpression and p53 silencing (**Figure 4C**). Meanwhile, RT-qPCR results indicated that GAS5 overexpression weakened the expression of TERT mRNA, but that GAS5 silencing increased the TERT mRNA expression. GAS5 overexpression combined with p53 silencing downregulated the TERT mRNA expression (**Figure 4D**).

As demonstrated by CCK-8 and colony formation assays, GAS5 overexpression reduced cell proliferation and colony formation but the opposite results were noted in response to GAS5 silencing. In addition, treatment with GAS5 + sh-p53 inhibited cell proliferation and colony formation (**Figures 4E, F** **)**. In addition, GAS5 overexpression arrested more cells in the G0/G1 phase and reduced cells arrested in the S phase, while there was no difference in the G2/M phase-arrested cells. No changes were found upon simultaneous GAS5 overexpression and p53 silencing. However, silencing GAS5 alone arrested fewer cells in the G0/G1 phase and increased the proportion of cells arrested in the S phase, accompanied by restored G2/M phase-arrested cells (**Figure 4G**). Further, cells overexpressing GAS5 showed increased Bax and cleaved caspase-3 expression, yet decreased Bcl-2 expression, while opposite effects were observed upon silencing of GAS5. Conversely, both GAS5 overexpression and p53 silencing led to augmented Bax and cleaved caspase-3 expression yet diminished Bcl-2 expression (**Figure 4H**). These findings suggest that GAS5 could facilitate p53 stability to inhibit telomerase activity and proliferation but suppress apoptosis of LSCC cells.

GAS5 has been reported to directly bind to p53 and p300, thus stabilizing the p53–p300 interaction (22). In order to validate the mechanism by which GAS5 regulates the stability of p53, we performed Co-IP assays to detect the effect of GAS5 on the role of p300 in stabilizing p53. As depicted, compared with Ad-control, p300 antibody could immunoprecipitate p53 from cell extracts upon GAS5 overexpression, which was reversed by further treatment with p300 inhibitor C646 (**Figure 4I**). Following GAS5 silencing, p300 antibody immunoprecipitated p53 from cell extracts was reduced, which was further reduced by further treatment with p300 inhibitor C646 (**Figure 4J**). In addition, p53 antibody could co-immunoprecipitate both p53 and p300 from cell extracts upon GAS5 overexpression, while GAS5 silencing resulted in opposite results (**Supplementary Figure S4A**). Meanwhile, the results of RIP assay showed that GAS5 was enriched in the p300 antibody (**Figure 4K**). RT-qPCR data exhibited a decline of TERT mRNA expression in the presence of p53 overexpression (**Figure 4L**). PCR-ELISA results showed that the telomerase activity could be decreased by p53 overexpression (**Figure 4M**). Collectively, GAS5 could stabilize p53 by recruiting p300 to bind with p53, thus repressing telomerase activity and proliferation of LSCC cells.

### YY1 Suppresses p53 Stability to Enhance Telomerase Activity and Proliferation of LSCC Cells

**Figure 5:**
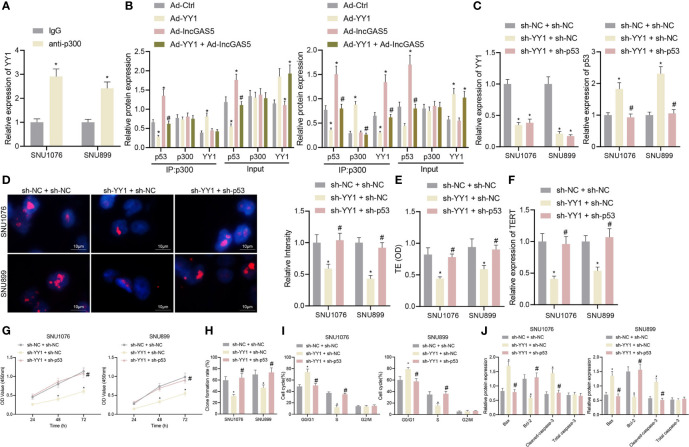
YY1 facilitates telomerase activity and proliferation but inhibits apoptosis of LSCC cells through p53 stability suppression. SNU1076 and SNU899 cells were transfected with sh-YY1 or in combination with sh-p53. **(A)** ChIP detects the interaction between YY1 and p300, * indicates *p* < 0.05 compared with IgG. **(B)** Co-IP detects the interaction between p300 and p53, * indicates *p* < 0.05 compared with Ad-Ctrl, # indicates *p* < 0.05 compared with Ad-YY1. **(C)** The expression of YY1 and p53 stability in cells determined by RT-qPCR. **(D)** Telomere length of cells measured by Q-FISH (the left). Blue indicates nucleus, and red indicates telomere. **(E)** Telomerase activity in cells detected by PCR-ELISA. **(F)** mRNA expression of TERT in cells determined by RT-qPCR. **(G)** Cell proliferation measured by CCK-8 assay. **(H)** Colony formation of cells measured by colony formation assay. **(I)** Cell cycle distribution measured by flow cytometry. **(J)** Western blot analysis of Bax, Bcl-2 and cleaved caspase-3 proteins in cells, normalized to GAPDH, * indicates *p* < 0.05 compared with sh-NC + sh-NC, # indicates *p* < 0.05 compared with sh-YY1 + sh-NC. Data are shown as mean ± standard deviation of three technical replicates. Data between two groups were compared by unpaired *t*-test while those among multiple groups were compared by one-way ANOVA with Tukey’s *post-hoc* tests. Bonferroni-corrected two-way ANOVA was applied for data comparison at various time points.

Having identified the interaction between YY1 and GAS5, GAS5 and p53, we continued to explore the possible correlation between YY1 and p53. We first verified whether p300 was enriched in YY1 by ChIP assay, and the results showed that compared with IgG, p300 antibody was significantly enriched in YY1 (**Figure 5A**). Co-IP results suggested that the interaction between p300 and p53 was decreased by overexpression of YY1, which was rescued by GAS5 overexpression. Compared with YY1 overexpression alone, their interaction was enhanced after dual overexpression of YY1 and GAS5, which was due to the increased recruitment of p300 by GAS5 after overexpression of GAS5, which reduced the binding of p300 and YY1 (**Figure 5B** and **Supplementary Figure S4B**).

Next, in order to further determine the regulatory relationship between YY1 and p53, we intervened the expression of YY1 and p53 at the same time. The expression of YY1 was decreased while stability of p53 protein was elevated in addition to reduced telomere length following YY1 silencing. However, dual silencing of YY1 and p53 recovered the p53 stability and the telomere length (**Figures 5C, D** **)**. In addition, PCR-ELISA revealed a decrease of telomerase activity in the presence of YY1 silencing, while opposite results were observed in the presence of simultaneous silencing of YY1 and p53 (**Figure 5E**). Meanwhile, YY1 silencing led to a reduction of the mRNA expression of TERT but further p53 silencing increased the TERT mRNA expression (**Figure 5F**). These data indicate that YY1 promotes telomere expression and activity of LSCC cells by downregulating p53.

As displayed in **Figures 5G, H**, YY1 silencing reduced cell proliferation and colony formation, which were restored in response to simultaneous silencing of YY1 and p53. Besides, there were more cells arrested in the G0/G1 phase and fewer cells arrested in the S phase, while the cells arrested in the G2/M phase did not differ following YY1 silencing. By contrast, the cell cycle was recovered in response to simultaneous silencing of YY1 and p53 (**Figure 5I**). At the same time, cells with YY1 silencing presented with increased Bax and cleaved caspase-3 expression, yet decreased Bcl-2 protein expression. Conversely, both silencing of YY1 and p53 recovered expression of these factors (**Figure 5J**). In conclusion, YY1 reduced the stability of p53 by inhibiting GAS5, and also directly interacted with p300 to inhibit interaction of p300 and p53 thus to reduce the stability of p53, thus promoting telomerase activity and proliferation but inhibiting apoptosis of LSCC cells.

### YY1 Facilitates the Tumor Growth by Inhibiting p53 Stability *In Vivo*

**Figure 6:**
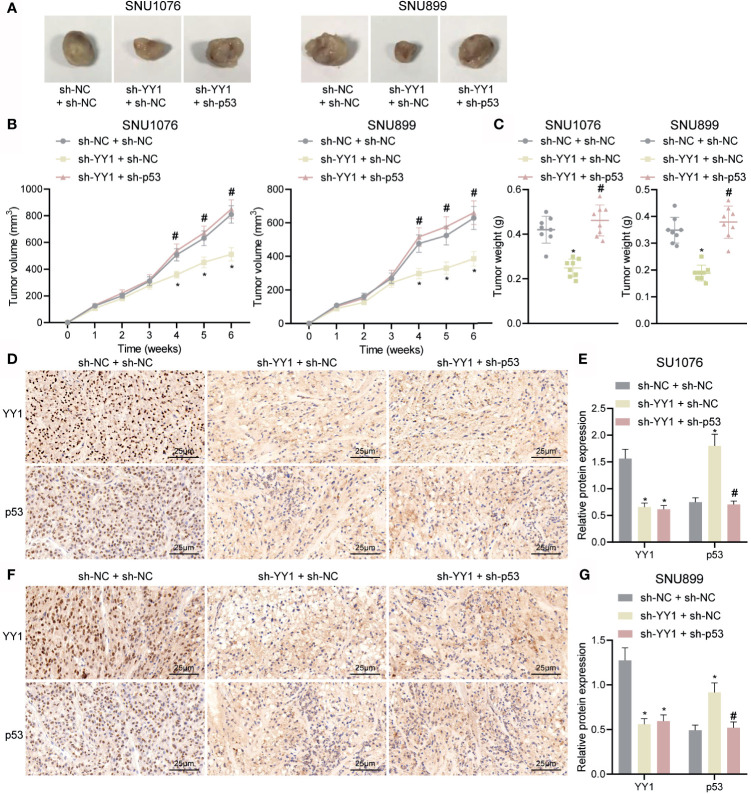
YY1 promotes tumor growth of LSCC *via* p53 stability suppression in *vivo*. Mice were injected with SNU1076 and SNU899 cells transfected with sh-YY1 + sh-NC or sh-YY1 + sh-p53. **(A)** Representative images showing xenografts in nude mice. **(B)** Tumor volume of mice. **(C)** Tumor weight of mice. **(D)** Immunohistochemical staining of YY1 and p53 proteins in tumor tissues of mice injected with SNU1076 cells. **(E)** Quantitation of panel **(D)**. **(F)** Immunohistochemical staining of YY1 and p53 proteins in tumor tissues of mice injected with SNU899 cells. **(G)** Quantitation of panel **(F)** * indicates *p* < 0.05 compared with sh-NC + sh-NC, # indicates *p* < 0.05 compared with sh-YY1 + sh-NC. Data are shown as mean ± standard deviation of three technical replicates. Data among multiple groups were compared by one-way ANOVA with Tukey’s *post-hoc* tests while those among multiple groups were compared by one-way ANOVA with Tukey’s *post-hoc* tests. Bonferroni-corrected repeated measures ANOVA was applied for data comparison at various time points. n = 8 for mice upon each treatment.

Finally, we sought to characterize the effect of YY1 on LSCC tumor growth by regulating p53 stability *in vivo*. As shown in **Figures 6A–C**, compared with sh-NC + sh-NC tumor volume and weight were decreased in mice treated with sh-YY1 + sh-NC, while combined treatment with sh-YY1 + sh-p53 enhanced the tumor volume and weight compared with sh-YY1 + sh-NC. In addition, immunohistochemical staining results suggested a decline of YY1 expression and an increase of p53 stability in the tumor tissues of mice treated with sh-YY1 + sh-NC compared with sh-NC + sh-NC. However, simultaneous silencing of YY1 and p53 weakened the stability of p53 in tumor tissues of mice compared with silencing of YY1 alone (**Figures 6D–G**). The aforementioned data supported the promoting effect of YY1 on the tumor growth of LSCC by reducing p53 stability *in vivo*.

## Discussion

The mortality rate of LSCC is on the rise despite recent advances in treatment modalities (23). Mounting evidence has indicated the presence of increased YY1 levels in various types of tumors and supported its crucial role for tumor cell proliferation and metastasis (24). In the current study, we aimed to explore the underlying mechanisms of YY1 in the pathogenesis of LSCC. Our findings suggested that YY1 could repress expression of GAS5 by binding to the GAS5 promoter, inducing a decline of p53 stability. Additionally, YY1 can directly interact with p300 to decrease the stability of p53, and thus elevate hTERT transcription and promote the LSCC progression.

Initially, the current study unveiled that YY1 was abundantly expressed in the LSCC cell lines and could potentiate LSCC cell proliferation and promote tumor growth. There is no an abundance of evidence that YY1 is overexpressed in multiple cancer types and that increased YY1 levels correlate with stronger malignant characteristics in many cancers. For instance, a significant increase of YY1 expression was upregulated in both thyroid cancer cells and tissues (25). YY1 also showed augmented expression in clinical tissue samples of oral cancer patients compared to adjacent normal tissues; its upregulation demonstrated pro-proliferative and pro-metastatic roles in oral cancer (26). Abundant expression of YY1 has been detected in laryngeal cancer tissue samples of patients with lymphatic metastasis, and it can enhance proliferation and migration of laryngeal cancer cells by directly inhibiting MYCT1 (8). Therefore, targeting YY1 may be a potential therapeutic strategy for metastatic LSCC.

GAS5 is found to be downregulated in LSCC tissues and cell lines, and conversely, its ectopic expression results in a marked inhibition of cell proliferation through the negative regulation of miR-21 (13), which concurs with our present results. In addition, the majority of lncRNAs exhibit a correlation with telomere length (27), whereby lncRNAs contribute to inhibition of hTERT expression (28). TERT extends telomere length to an extent that correlates with the proliferation capacity of both normal and cancerous cells, and telomerase activation upon TERT production widely occurs in human malignant cells, which is essential for malignant transformation (29). The presence of co-activation of TERT and YY1 was determined in a subset of gastric tumors (30). Meanwhile, YY1 adversely modulates GAS5 expression by binding to the GAS5 promoter and thus represses its transcription in neurons following cerebral ischemia/reperfusion injury (31). Based on these findings in a broad range of settings, we are convinced that YY1 can enhance telomerase activity and proliferation of LSCC cells by negatively regulating GAS5.

In addition, the current study demonstrated that GAS5 suppressed telomerase activity and proliferation of LSCC cells by stabilizing p53. Similarly, GAS5 has been recently revealed to interact with and stabilize p53 protein, depending on the exon 12 in response to gastric cancer (32). Moreover, GAS5 can bind to p53 and p300 in a direct manner, and stabilize the interaction between p53 and p300 by upregulating the p53 downstream target genes in vascular smooth muscle cells during vascular remodeling (22). At the same time, increased p53 expression following TUG1 downregulation markedly attenuates the proliferation, cell cycle progression, migration, and invasion of LSCC cells (33). In addition, cardiac senescence induced by doxorubicin occurs concomitantly with decreased cellular proliferation, increased expression of p53, and decreased telomere length and telomerase activity (34).

A previously published investigation showed that YY1 blunts the interaction between p53 and the co-activator p300, as well as disrupting p300-dependent acetylation and stabilization of p53 protein; furthermore, YY1 inhibits the accumulation of p53 in response to genotoxic stress (35). Meanwhile, a recent work confirmed the interaction between YY1 and p300, whereby p300 antibody can immunoprecipitate YY1 from cell extracts (36). Besides, it has been well-documented that YY1 has the ability to promote tumor growth through reductions in p53 levels (37). Considering these experimental data, we reasoned that YY1 can directly interact with p300 to reduce the stability of p53, thus enhancing telomerase activity and proliferation of LSCC cells *in vitro* as well as facilitating tumor growth *in vivo*.

**Figure 7:**
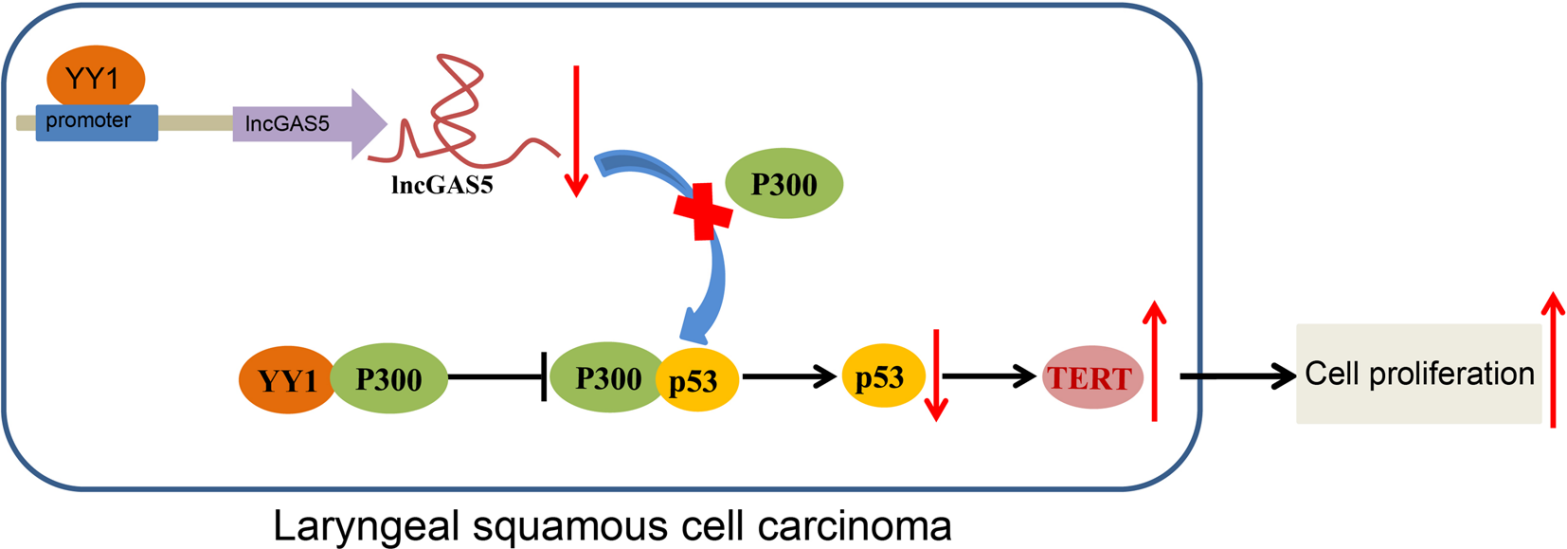
Schematic diagram of the mechanism by which YY1 affects LSCC. YY1 inhibits the expression of GAS5 by binding to the GAS5 promoter, thereby reducing the stability of p53. By this mechanism, hTERT transcription is increased and the occurrence of LSCC is promoted. On the other hand, YY1 can directly interact with p300 to affect the stability of p53, thus upregulating hTERT transcription and promoting the occurrence of LSCC.

In conclusion, the current study provides evidence that YY1 can potentially block GAS5-dependent stability of p53 protein and cause the inhibition of telomerase activity and proliferation of LSCC cells. Also, YY1 can directly interact with p300 to reduce the stability of p53, thus upregulating TERT expression and promoting the occurrence of LSCC (**Figure 7**). These findings strengthen our understanding of the mechanisms of LSCC and may eventually serve as the basis for a new prognostic marker for the treatment of LSCC. However, future studies should employ specimens from a large population of LSCC-diagnosed patients for in-depth analysis. Additionally, due to the lack of available literature regarding the role of GAS5 in telomerase activity, more research is warranted aiming to support translation of present findings into clinical practice.

## Data Availability Statement

The original contributions presented in the study are included in the article/**Supplementary Material**. Further inquiries can be directed to the corresponding author.

## Ethics Statement

The animal study was reviewed and approved by Gansu Provincial Hospital.

## Author Contributions

XW conceived and designed research. FL and TZ performed experiments and analyzed data. XJ and XX prepared figures. XW and FL drafted manuscript. XJ and CK edited and revised manuscript. All authors contributed to the article and approved the submitted version.

## Funding

This study was supported by Key project of the National Scientific Research Cultivation Plan of Gansu Provincial Hospital (19SYPYA-13).

## Conflict of Interest

The authors declare that the research was conducted in the absence of any commercial or financial relationships that could be construed as a potential conflict of interest.

## Publisher’s Note

All claims expressed in this article are solely those of the authors and do not necessarily represent those of their affiliated organizations, or those of the publisher, the editors and the reviewers. Any product that may be evaluated in this article, or claim that may be made by its manufacturer, is not guaranteed or endorsed by the publisher.

## Supplementary Material

Supplementary Figure 1 Adverse correlation between YY1 and GAS5. **(A)** Co-expression analysis of YY1 and GAS5 using the MEM database. **(B)** Correlation between YY1 and GAS5 in LSCC using the GEPIA database.

Supplementary Figure 2 YY1 boosts telomerase activity and proliferation but reduces apoptosis of LSCC SNU899 cells by suppressing GAS5. **(A)** Subcellular localization of YY1 (green) and GAS5 (red) determined by FISH assay. Blue indicates DAPI staining. **(B)** ChIP-qPCR was used to detect the binding of YY1 to GAS5 promoter. Primers were designed for P1 and P2 upstream of YY1 and C1 and C2 downstream of YY1. qPCR detected ChIP enriched DNA, * indicates *p* < 0.05 compared with sh-NC + ASO-NC. **(C)** GAS5 expression determined by RT-qPCR in SNU1076 cells transfected with sh-YY1, * indicates *p* < 0.05 compared with sh-NC. **(D)** Knockdown efficiency of GAS5 confirmed by RT-qPCR in SNU1076 cells, * indicates *p* < 0.05 compared with ASO-NC. **(E)** Telomere length of cells measured by Q-FISH. Blue indicates nucleus, red indicates telomere, and green indicates centromere. **(F)** Telomerase activity in cells detected by PCR-ELISA. **(G)** Western blot analysis of YY1 and TERT proteins in SNU1076 cells, normalized to GAPDH. **(H)** mRNA expression of TERT and GAS5 in SNU1076 cells determined by RT-qPCR. **(I)** SNU1076 cell proliferation measured by CCK-8 assay. **(J)** Colony formation of SNU1076 cells measured by colony formation assay. **(K)** SNU1076 cell cycle distribution measured by flow cytometry. **(L)** Western blot analysis of Bax, Bcl-2 and cleaved caspase-3 proteins in SNU1076 cells, normalized to GAPDH, * indicates *p* < 0.05 compared with sh-NC + ASO-NC. Data are expressed as mean ± standard error of the mean. Data are shown as mean ± standard deviation of three technical replicates. Data between two groups were compared by unpaired *t*-test while those among multiple groups were compared by one-way ANOVA with Tukey’s *post-hoc* tests. Bonferroni-corrected two-way ANOVA was applied for data comparison at various time points.

Supplementary Figure 3 Verification of the correctness of TERT antibody. **(A)** RT-qPCR used to detect the expression level of TERT mRNA, * indicates *p* < 0.05 compared with NC. **(B)** The expression of TERT protein detected by Western blot.

Supplementary Figure 4 Co-IP results. **(A)** The interaction between GAS5, p300 and p53. **(B)** interaction between p300 and p53 after overexpression of YY1 and cGAS5.

Supplementary Figure 5 Representative images of FISH and Q-FISH assays. A, Subcellular localization of YY1 (green) and GAS5 (red) determined by FISH assay. Blue indicates DAPI staining. B, Telomere length of cells measured by Q-FISH. Blue indicates nucleus, red indicates telomere, and green indicates centromere.
