# Supplementary figures and images for: YY1 Promotes Telomerase Activity and Laryngeal Squamous Cell Carcinoma Progression Through Impairment of GAS5-Mediated p53 Stability
Source: Front Oncol. 2021 Aug 23;11:692405. doi: 10.3389/fonc.2021.692405 (PMC8421032; doi:10.3389/fonc.2021.692405)

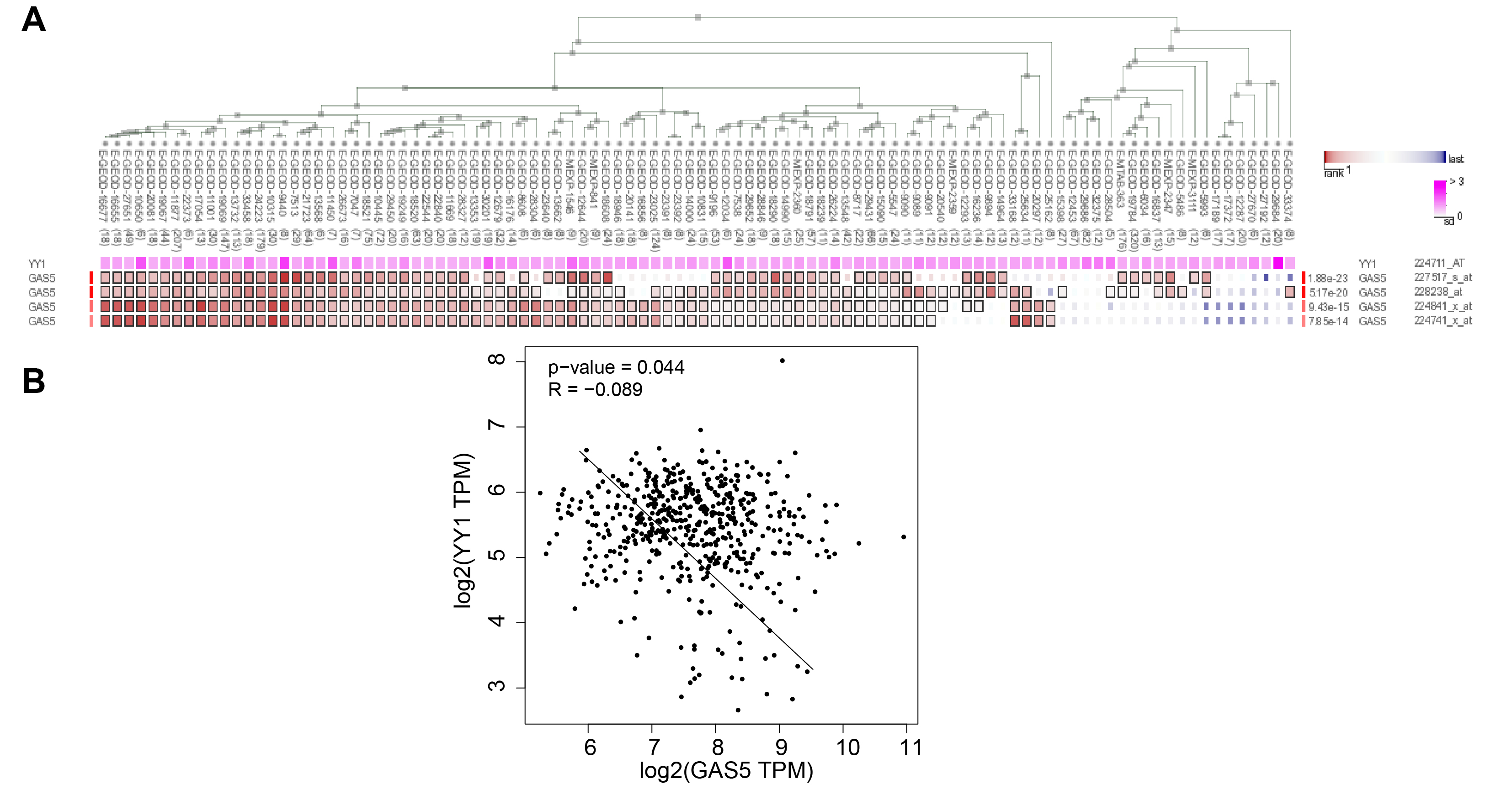

Supplement: Supplementary Figure 1 — Adverse correlation between YY1 and GAS5. (A) Co-expression analysis of YY1 and GAS5 using the MEM database. (B) Correlation between YY1 and GAS5 in LSCC using the GEPIA database. [file Image_1.jpeg]

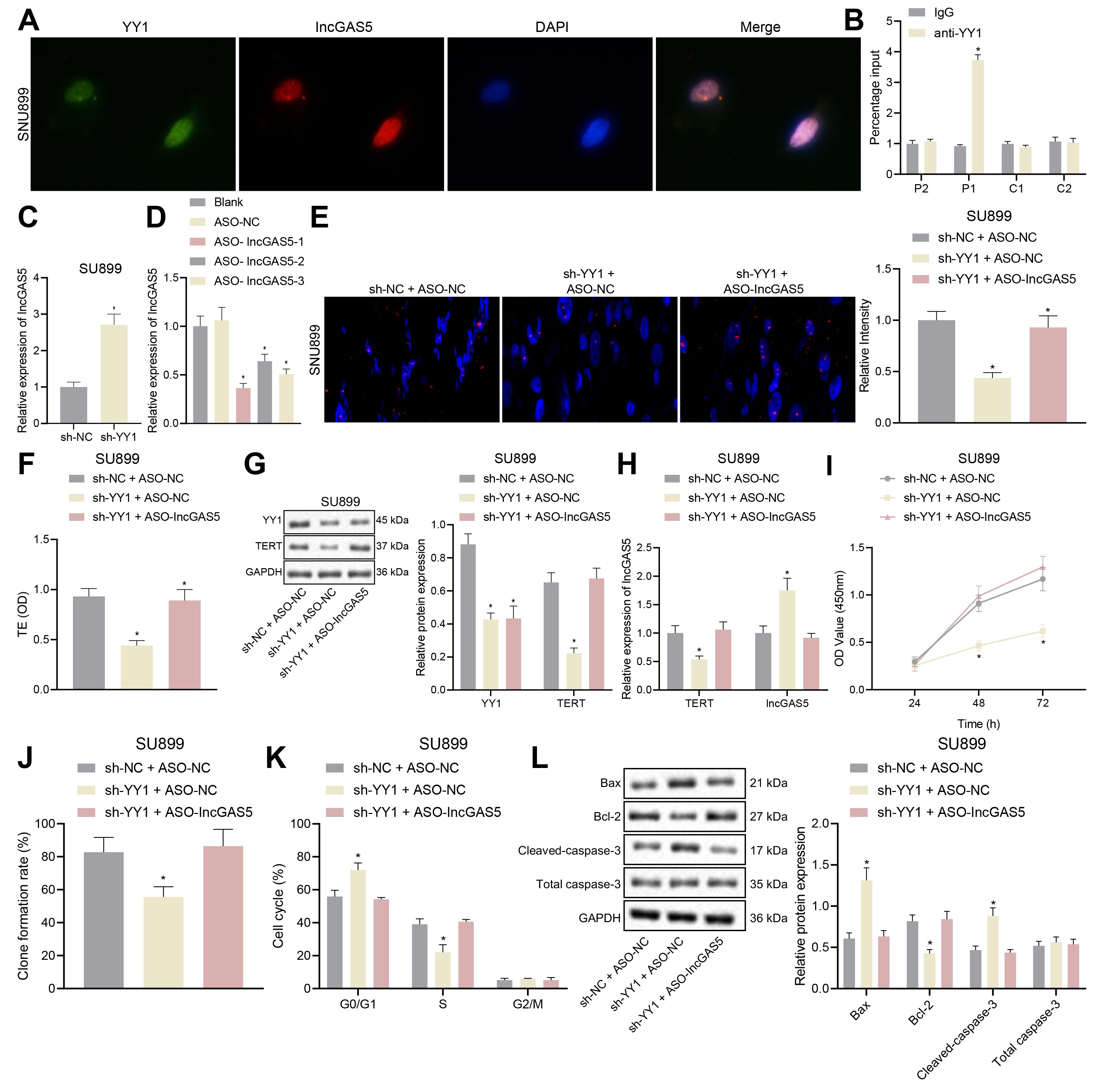

Supplement: Supplementary Figure 2 — YY1 boosts telomerase activity and proliferation but reduces apoptosis of LSCC SNU899 cells by suppressing GAS5. (A) Subcellular localization of YY1 (green) and GAS5 (red) determined by FISH assay. Blue indicates DAPI staining. (B) ChIP-qPCR was used to detect the binding of YY1 to GAS5 promoter. Primers were designed for P1 and P2 upstream of YY1 and C1 and C2 downstream of YY1. qPCR detected ChIP enriched DNA, * indicates p < 0.05 compared with sh-NC + ASO-NC. (C) GAS5 expression determined by RT-qPCR in SNU1076 cells transfected with sh-YY1, * indicates p < 0.05 compared with sh-NC. (D) Knockdown efficiency of GAS5 confirmed by RT-qPCR in SNU1076 cells, * indicates p < 0.05 compared with ASO-NC. (E) Telomere length of cells measured by Q-FISH. Blue indicates nucleus, red indicates telomere, and green indicates centromere. (F) Telomerase activity in cells detected by PCR-ELISA. (G) Western blot analysis of YY1 and TERT proteins in SNU1076 cells, normalized to GAPDH. (H) mRNA expression of TERT and GAS5 in SNU1076 cells determined by RT-qPCR. (I) SNU1076 cell proliferation measured by CCK-8 assay. (J) Colony formation of SNU1076 cells measured by colony formation assay. (K) SNU1076 cell cycle distribution measured by flow cytometry. (L) Western blot analysis of Bax, Bcl-2 and cleaved caspase-3 proteins in SNU1076 cells, normalized to GAPDH, * indicates p < 0.05 compared with sh-NC + ASO-NC. Data are expressed as mean ± standard error of the mean. Data are shown as mean ± standard deviation of three technical replicates. Data between two groups were compared by unpaired t-test while those among multiple groups were compared by one-way ANOVA with Tukey’s post-hoc tests. Bonferroni-corrected two-way ANOVA was applied for data comparison at various time points. [file Image_2.jpeg]

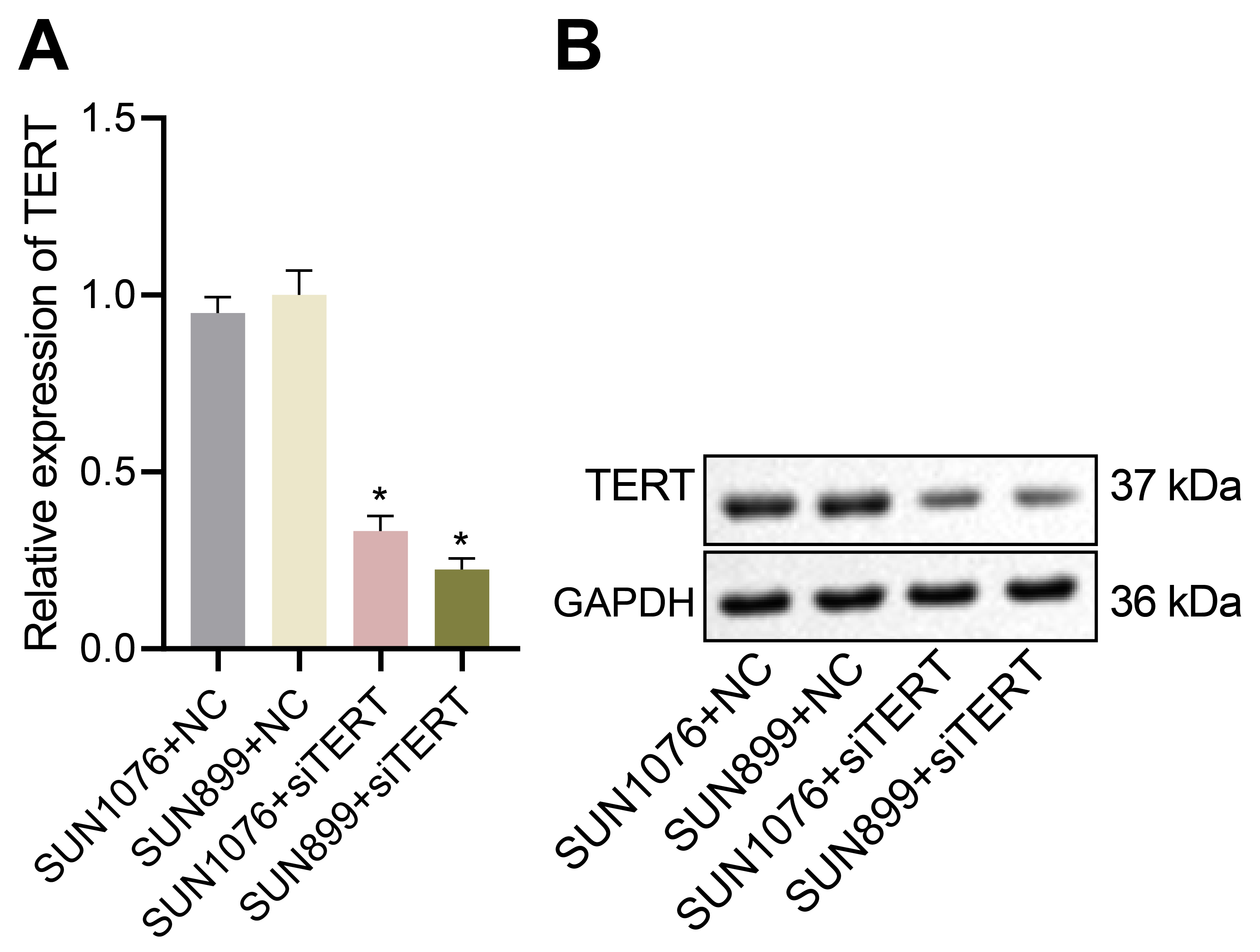

Supplement: Supplementary Figure 3 — Verification of the correctness of TERT antibody. (A) RT-qPCR used to detect the expression level of TERT mRNA, * indicates p < 0.05 compared with NC. (B) The expression of TERT protein detected by Western blot. [file Image_3.jpeg]

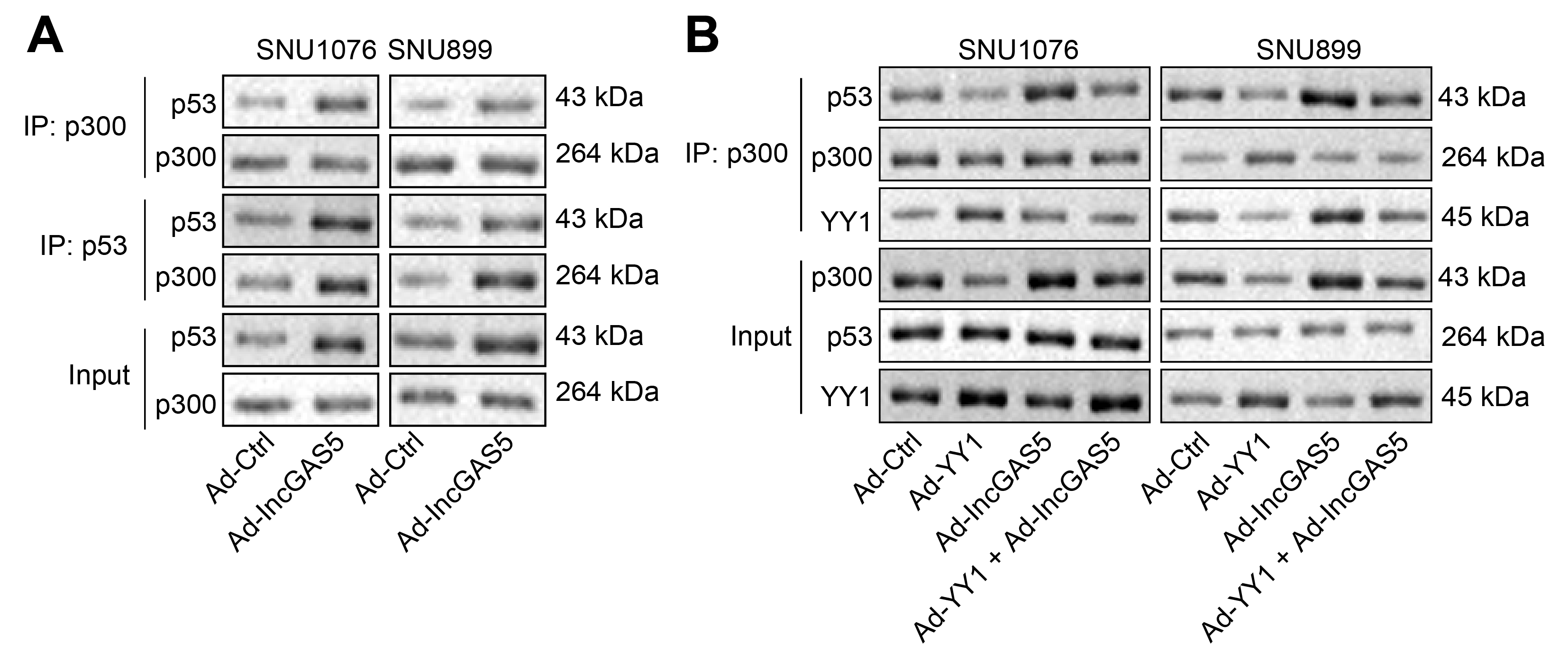

Supplement: Supplementary Figure 4 — Co-IP results. (A) The interaction between GAS5, p300 and p53. (B) interaction between p300 and p53 after overexpression of YY1 and cGAS5. [file Image_4.jpeg]

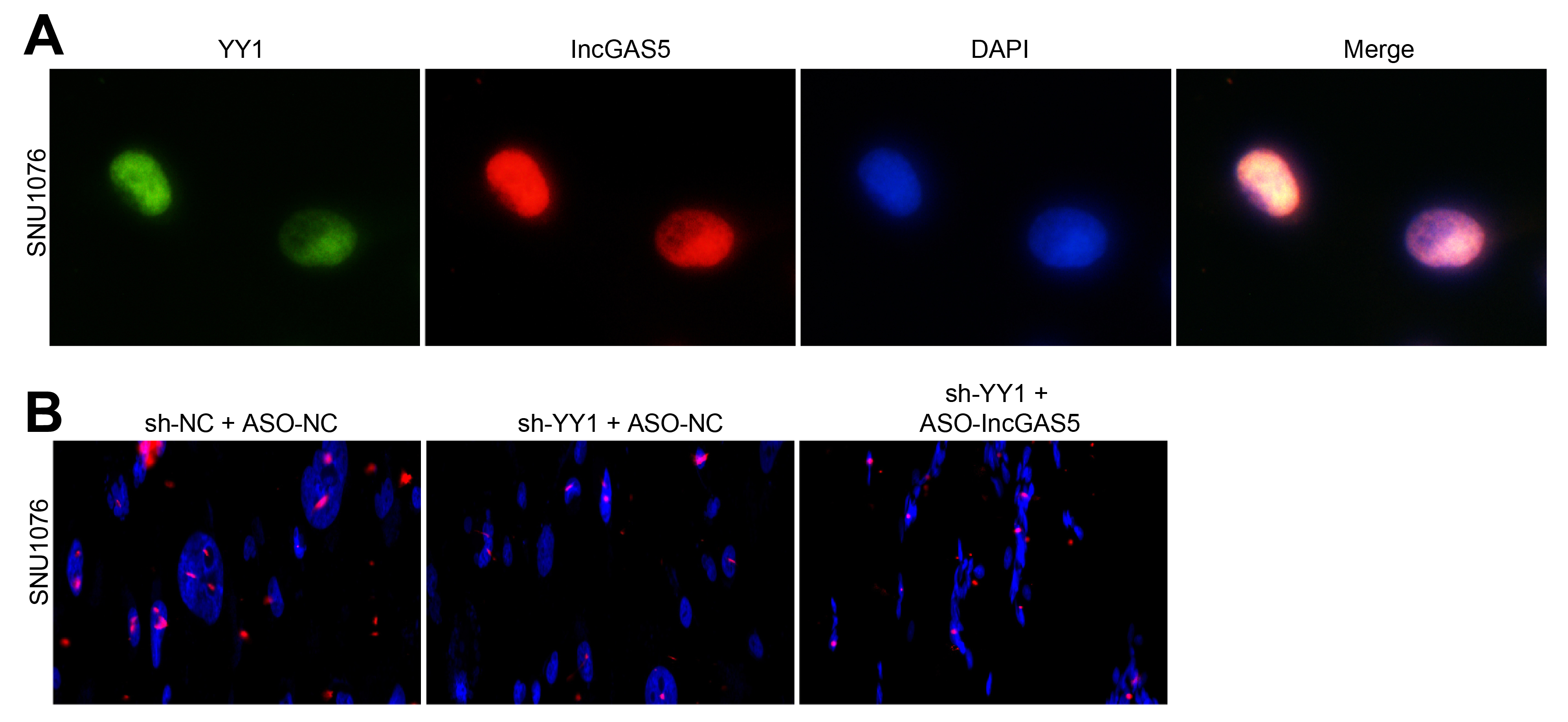

Supplement: Supplementary Figure 5 — Representative images of FISH and Q-FISH assays. A, Subcellular localization of YY1 (green) and GAS5 (red) determined by FISH assay. Blue indicates DAPI staining. B, Telomere length of cells measured by Q-FISH. Blue indicates nucleus, red indicates telomere, and green indicates centromere. [file Image_5.jpeg]
