# Supplementary material for: YY1 Promotes Telomerase Activity and Laryngeal Squamous Cell Carcinoma Progression Through Impairment of GAS5-Mediated p53 Stability
Source: Front Oncol. 2021 Aug 23;11:692405. doi: 10.3389/fonc.2021.692405 (PMC8421032; doi:10.3389/fonc.2021.692405)
Supplement: Supplementary file 6 [file Table_1.doc]

**Table S1** Primer sequences for RT-qPCR

| Gene | Sequence |  |
| --- | --- | --- |
| YY1 | F: 5’-ACGGCTTCGAGGATCAGATTC-3’ | |
|  | R: 5’-TGACCAGCGTTTGTTCAATGT-3’ | |
| GAS5 | F: 5’-TATGGTGCTGGGTGCAGAT-3’ | |
|  | R: 5’-CCAATGGCTTGAGTTAGGCTT-3’ | |
| TERT | F: 5’-CGGAAGAGTGTCTGGAGCAA-3’ | |
|  | R: 5’-CTCCCACGACGTAGTCCATG-3’ | |
| p53 | F: 5’-CAGCACATGACGGAGGTTGT-3’ | |
|  | R: 5’-TCATCCAAATACTCCACACGC-3’ | |
| p300 | F: 5’-ACCAGGAATGACTTCTAGTTTGA-3’ | |
|  | R: 5’-TTCCCTGTGATGGGAACTGA-3’ | |
| GAPDH | F: 5’-CTGGGCTACACTGAGCACC-3’ | |
|  | R: 5’-AAGTGGTCGTTGAGGGCAATG-3’ | |
