# Supplementary material for: YY1 Promotes Telomerase Activity and Laryngeal Squamous Cell Carcinoma Progression Through Impairment of GAS5-Mediated p53 Stability
Source: Front Oncol. 2021 Aug 23;11:692405. doi: 10.3389/fonc.2021.692405 (PMC8421032; doi:10.3389/fonc.2021.692405)
Supplement: Supplementary file 7 [file Table_2.doc]

**Table S2 Core degree ranking of genes in the protein interaction network**

| Rank | Gene | Degree | Rank | Gene | Degree |
| --- | --- | --- | --- | --- | --- |
| 1 | BRCA1 | 28 | 16 | MAZ | 15 |
| 2 | KAT2B | 27 | 19 | CBX3 | 14 |
| 3 | YY1 | 26 | 19 | FOXP3 | 14 |
| 4 | E2F1 | 24 | 19 | IRF3 | 14 |
| 4 | STAT1 | 24 | 19 | PML | 14 |
| 6 | FOXM1 | 23 | 19 | STAT2 | 14 |
| 6 | KDM1A | 23 | 19 | TP63 | 14 |
| 6 | PPARG | 23 | 19 | WHSC1 | 14 |
| 6 | PRKDC | 23 | 26 | CENPA | 13 |
| 10 | HIF1A | 22 | 26 | TBL1XR1 | 13 |
| 11 | CEBPB | 21 | 28 | FOXK1 | 12 |
| 12 | USF1 | 19 | 28 | RNF2 | 12 |
| 13 | MYBL2 | 16 | 30 | HSF1 | 11 |
| 13 | SNAI2 | 16 | 31 | HOXB7 | 9 |
| 13 | TEAD4 | 16 | 32 | HOXC11 | 5 |
| 16 | ARNTL | 15 | 32 | HOXC9 | 5 |
| 16 | E2F7 | 15 | 34 | EHF | 4 |
